# Inferring Adaptation at Community Composition Level From the Relationship Between Succession Rate and Global Change Intensity

**DOI:** 10.1111/1758-2229.70426

**Published:** 2026-09-30

**Authors:** Tingting Li, Rong Mao, Yang Zhang, Ximei Zhang

**Affiliations:** ^1^ College of Forestry Jiangxi Agricultural University Nanchang China; ^2^ Key Laboratory of Dryland Agriculture, Ministry of Agriculture, Institute of Environment and Sustainable Development in Agriculture Chinese Academy of Agricultural Sciences Beijing China; ^3^ Experimental Centre of Forestry in North China Chinese Academy of Forestry Beijing China; ^4^ State Key Laboratory of Efficient Utilization of Agricultural Water Resources CAU/CAAS Beijing China

**Keywords:** deterministic process, nitrogen deposition, nitrogen enrichment, soil microbial community, stochastic process, succession rate

## Abstract

In response to anthropogenic environmental changes, biological communities often undergo compositional changes, which are traditionally thought to represent adaptation. However, this hypothesis and its mechanism remain surprisingly underexplored. In this study, we conducted a 10‐year nitrogen deposition experiment in the Eurasian steppe, manipulating nine intensities under two management strategies (fencing or mowing). As the intensity increased from 0–20 to 50 g N m^−2^ yr^−1^, the succession rates of both plant and soil microbial communities first increased but then decreased, implying that high intensities of N deposition exceed the capacity of community adaptation. In addition, both the succession rate of the deterministic component of community compositional variation and the succession rate of soil physicochemical characteristics increased and then decreased with increasing N intensity. They were positively correlated, demonstrating that adaptation is primarily driven by deterministic ecological processes. Meanwhile, mowing promoted the plant community's adaptation, while fencing promoted adaptation of the microbial community, both by restraining stochastic processes. Overall, to enable biological communities to adapt compositionally to environmental changes, we should aim to limit the intensity of changes below certain thresholds, and implement reasonable strategies, whether deterministic (e.g., restoring soil physicochemical conditions) or stochastic (e.g., adding seeds of adaptive species).

## Graphical abstract

A 10‐year nitrogen addition experiment in Eurasian steppe reveals that plant and microbial community succession rates first increase then decrease with rising N intensity, indicating an adaptive threshold. Deterministic processes driven by soil physicochemical changes dominate this response, with management strategies modulating adaptation via stochastic processes.

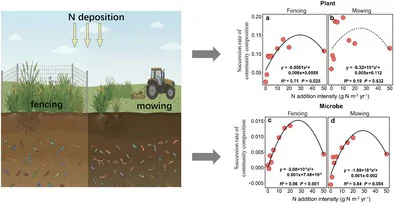

## 1 Introduction

A central goal in current ecology is to investigate the relationship between anthropogenic environmental changes and organisms within various ecosystems (Hautier et al. 2015). There are two opposite aspects to that relationship: one is that environmental changes may filter out the species whose fitness is low (Cadotte and Tucker 2017; Castellanos et al. 2019; Le Bagousse‐Pinguet et al. 2017; Renault et al. 2018); the other is that adaptation to these changes can occur through processes operating at three levels. Firstly, physiological adaptation may occur quickly via phenotypic plasticity, on a temporal scale shorter than one generation. Secondly, on the scale of several to dozens of generations, community‐level adaptation may occur through shifts in species composition, which may happen concurrently with physiological adaptations. Finally, on an even longer evolutionary temporal scale, genetic adaptation may arise. In the past several decades, the adjustments at physiological and genetic levels have been detected in many studies (e.g., Belay et al. 2024; Ho and Zhang 2018), with a few examined functionally or experimentally to confirm adaptation. Adjustments at the community compositional level have also been observed and taken as evidence for adaptation in many studies (Chang et al. 2019; Liang et al. 2020; Zhou et al. 2014). In modern ecology, directional compositional changes under chronic anthropogenic pressure (e.g., warming, nitrogen enrichment) are increasingly conceptualized within the framework of ecological succession (Guo et al. 2018; Liang et al. 2020; Li and Shipley 2018). Accordingly, the pace of these directional changes—the succession rate—is a key metric for quantifying adaptive compositional turnover. Yet, as far as we know, far fewer studies have experimentally tested whether that variation in community composition leads to full adaptation to environmental changes.

Theoretically speaking, different species in the species pool of a given ecosystem occupy distinct ecological niches, that is, they have different optimal environmental conditions (Silvertown 2004). After environmental changes occur, the composition of the community will change accordingly, that is, some relatively adapted species will be favoured, while some others will be selected against (Keddy 1992). However, the number of species in the species pool is limited, so the range of environmental changes to which community composition can adapt through compositional shifts is also limited. Therefore, as the intensity of global change increases, the rate of community temporal change may exhibit a non‐linear pattern of initially increasing and then decreasing. If the global change intensity is very low, adaptive adjustment at the level of community composition can occur through slight changes in species composition, resulting in a slight increase in the rate of community temporal change. As the intensity increases, community composition is expected to shift more strongly, leading to a significant increase in the rate of community temporal change. However, if the intensity is so great that it exceeds the adaptation range of all species, the community reaches a non‐adaptive stable state in a short time. This stability is not successful adaptation but rather a state of constrained adaptation resulting from the depletion of replaceable species. Consequently, subsequent community changes are minimal, and the overall temporal turnover rate decreases. In other words, adaptation at the community compositional level is constrained below a certain threshold of environmental change intensity. Thus, based on the relationship between the rate of community temporal change (i.e., community succession rate) and global change intensity, we can infer whether temporal community compositional variation under a given global change intensity fully represents adaptation or not. Specifically, as the global change intensity increases from very low to a certain magnitude, if the community succession rate continues to increase, this would mean a greater rate of compositional adjustment, suggesting that the community is capable of adapting to that certain intensity. Conversely, if the succession rate begins to decrease, this would suggest that adaptation at the community level becomes constrained at that certain intensity.

To effectively maintain biodiversity and their ecosystem functions under environmental changes, we should further explore the mechanisms underlying adaptation at the community compositional level. Actually, both deterministic (e.g., ecological filtering and interspecific competition) and stochastic (e.g., random birth/death and dispersal/colonization) ecological processes are potential drivers of community compositional dynamics in all types of ecosystems (Mori et al. 2018; Nemergut et al. 2013; Zhou et al. 2014), and either may be promoted or restrained by anthropogenic global changes (Alberti et al. 2017; Liang et al. 2020; Zhang et al. 2011; Zhang, Johnston, et al. 2016). Adaptation to a certain type of global change at the community composition level is a directional process, and so it is achievable by promoting deterministic ecological processes (e.g., selecting for the adaptive species) or restraining stochastic ecological processes (e.g., restricting the immigration/colonization of un‐adapted species). However, we do not know which of those two sets of processes constitutes the principal mechanism underlying compositional adaptation. In tandem, we want to know whether particular types of ecosystem management strategies could help to hasten the succession rate and thus enhance community adaptation to global changes. Moreover, what is the underlying mechanism operating (the promotion of deterministic processes vs. the restraint of stochastic processes) under a given management strategy?

In this study, we conducted a 10‐year nitrogen (N) deposition experiment with nine intensities (0–50 g N m^−2^ yr^−1^) under two grassland management strategies (fencing or mowing) in a typical steppe ecosystem of Inner Mongolia of China, which is representative of much of the Eurasian steppe region, floristically and ecologically (Li et al. 1988). We investigated both the plant community composition and the soil microbial biomarker (PLFA) profiles, aiming to answer three key questions. First, as the N deposition intensity increases from low to high, will the succession rates of plant and soil microbial community first increase but eventually decrease? That is, will adaptation at the community level occur at relatively low intensities but become constrained at high intensities? Second, are the adaptations at the community composition level achieved mainly by promoting deterministic processes or restraining stochastic processes? Third, does either management strategy affect the succession rate and thus the adaptation of these communities? And what is the pivotal mechanism involved (the promotion of deterministic processes vs. the restraint of stochastic processes)?

## 2 Material and Methods

### 2.1 Study Site and Experimental Design

This field study was part of a long‐term N deposition experiment conducted in a temperate steppe (116°14′ E, 43°13′ N), located in the Xilin River Basin of the Inner Mongolia Autonomous Region, China (Zhang et al. 2014). The experimental site has been fenced since 1999 to exclude large animals from grazing. Its mean annual precipitation is 348.5 mm, 81.8% of which occurs during the growing season (May to September), while its mean annual temperature is 0.9°C, with monthly means ranging from −21.3°C (January) to 19.7°C (August). The soil at the site is classified as Haplic Calcisols based on the FAO soil classification system, that corresponds to Calcic‐Orthic Aridisol based on the US soil classification system. The site's plant community is dominated by the perennial C3 rhizomatous grass *Leymus chinensis*, and the perennial C3 bunchgrass *Stipa grandis* (Zhang, Stevens, et al. 2016; Zhang, Johnston, et al. 2016). The ambient N deposition intensity in the field was < 1.5 g N m^−2^ yr^−1^ (Jia et al. 2014).

The field experiment was established during September 2008, by following a randomized complete block design that consisted of nine N addition intensities (0, 1, 2, 3, 5, 10, 15, 20, and 50 g N m^−2^ yr^−1^) under two grassland management strategies (fencing vs. mowing) (Zhang et al. 2013). The N addition was carried out once every month, by applying the equivalent of 1/12 of the whole year's intensity. From May to October, purified NH_4_NO_3_ was mixed with purified water, and this was sprinkled evenly onto each plot to simulate the wet N deposition. From November to next April, NH_4_NO_3_ was mixed with clean sand, and this was spread evenly by hand to simulate the dry N deposition. The sand was first passed through a < 1‐mm sieve, then dipped in hydrochloric acid, washed in purified water, and finally heated at 120°C for 24 h in an oven. All plots received the same amount of water (~0.14 mm) and sand (~3 kg) every year (Yang et al. 2023; Zhang et al. 2013). The mowing was performed with a mower at a 10‐cm height in late August (after reproduction had finished for most plant species), which simulated typical hay‐cutting management practices in this region; the harvested aboveground plants were removed immediately from each plot. Overall, there were 18 treatments in total, with six replicate plots per treatment.

### 2.2 Sample Collection and Measurement

Aboveground plant biomass was harvested in mid‐August, every year from 2010 through 2018, by using a 0.5 × 2 m rectangle per plot. This rectangle was randomly placed in each plot, ensuring no spatial overlap of quadrats among years, with it positioned at least 50 cm away from each plot's border to avoid any edge effects. All living vascular plants were clipped at the ground level and sorted according to species, then oven‐dried at 65°C for 48 h, and weighed. About 60% of the species in the 8 × 8 m^2^ plot were found in the 0.5 × 2 m^2^ subplot, demonstrating that this community is relatively even.

Four soil cores (10 cm in depth, 3.5 cm in diameter) were also collected randomly from each plot in mid‐August, every year from 2010 through 2018. The soil samples were passed through a 2‐mm sieve and then mixed to yield one composite sample per plot. The soil was then divided into two sets of subsamples: one, for the measurement of phospholipid fatty acid (PLFA), available N (NH_4_^+^‐N and NO_3_^−^‐N) and water content, was stored at 4°C; the other set, for the measurement of pH, was air‐dried. Soil available N content was determined using a Foss FIAstar 5000 flow injection auto‐analyzer (Foss Tecator, Denmark) after a 1 mol L^−1^ KCl extraction. Soil water content was determined as the weight loss after drying for 24 h at 105°C. Soil pH was measured with a pH metre (FE20‐FiveEasy) after shaking the soil in a suspension of distilled water (1:2.5 w/v).

PLFA analysis was used to characterize the soil microbial biomarker composition of the soil samples. Microbial lipids were extracted from soil with a one‐phase extraction mixture of chloroform, methanol, and phosphate buffer (v:v:v = 1:2:0.8), according to the procedure described by Bossio and Scow (1998). Phospholipids were separated from neutral lipids and glycolipids, by using a silica column (Supelco, USA (Supelco Inc., Bellefonte, PA)). After methylation of the polar lipids, PLFA methyl esters were separated and identified by gas chromatography (Agilent 6850, Agilent Technologies, USA), with a flame ionization detector on a capillary column ultra 2.4 μL injection, in the split mode (1:20). The individual PLFA methyl esters peaks were identified and quantified by using the MIDI Sherlock Microbial Identification System (MIDI, USA). The fatty acid 19:0 (Sigma Aldrich, USA) served as an internal standard. In this study, 20 common PLFA markers (Table S1) were used to represent different soil microbial groups. Among them, straight‐chain saturated fatty acids (14:0, 15:0, 16:0, 17:0, 18:0, 20:0) are non‐specific bacterial biomarkers, commonly used to estimate total microbial biomass; branched fatty acids (a15:0, a17:0, a17:1w9c, i14:0, i15:0, i16:0, i17:0) characterize Gram‐positive bacteria, which are often more resistant to nutrient stress; whereas monounsaturated fatty acids (16:1w9c, 17:1w8c, 18:1w5c, 20:1w9c) and cyclopropane fatty acids (e.g., cy17:0) primarily indicate Gram‐negative bacteria, which are more sensitive to changes in available carbon sources in the environment (Rappe‐George et al. 2017). 18:1ω9c is used as the signature biomarker for fungi. By analysing changes in these biomarkers, the response of soil microbial community structure and its ecological strategies under nitrogen addition and management practices can be assessed.

### 2.3 Succession Rate of Community Composition

Time‐decay relationships (TDRs) have been widely adopted to evaluate temporal community dynamics under long‐term environmental manipulations (Chen et al. 2015; Shade et al. 2013). For each treatment, the time‐decay relationships of plant and soil microbial community composition were quantified by linear regression between the logarithmic *β*‐similarities and logarithmic temporal distance (Guo et al. 2018; Liang et al. 2020; Wang et al. 2020): ln(*S*_*s*_) = *c*−vln(*T*), where *S*_*s*_ is the pairwise similarity in community composition, *T* is the time interval, *c* is the intercept, and the slope *v* is a measure of the temporal turnover rate of the community, namely the succession rate of community composition. To account for repeated temporal sampling of permanent plots, pairwise similarities within each treatment were calculated only between the same plot sampled in different years, thereby avoiding comparisons between different plots or blocks across years. The Bray–Curtis similarity metric was used in this study (Bray and Curtis 1957). The significance of *v* values was evaluated by a one‐sample *t*‐test between the original slope and bootstrapped slopes from random pairings of the original set (permuted 999 times) (Horner‐Devine et al. 2004; Zhou et al. 2008).

### 2.4 Succession Rate of the Deterministic and Stochastic Community Components

For each treatment, we first quantified the deterministic and stochastic components of community compositional variation between any 2 years, using a modified calculation framework originally developed by Zhang et al. (2011). In Zhang et al. (2011), untreated spatial plots were used as controls, and the mean compositional dissimilarity among control plots was defined as the reference baseline. This study focuses on temporal community dynamics rather than spatial treatment effects, hence a temporal reference framework was used. For any 2 years, denoted as year *t*_1_ and year *t*_2_ (*t*_1_ < *t*_2_), the community state in year *t*_1_ was treated as the temporal reference state for year *t*_2_. The 2 years did not need to be adjacent years, and all calculations were conducted within the same treatment, that is, within the same combination of N addition intensity and management strategy. Specifically, the *reference point* was calculated as the mean Bray–Curtis dissimilarity across all pairwise comparisons among replicate plots within the same treatment in year *t*_1_:

$$R_{t1}=\frac{2}{n\left(n-1\right)}\sum_{i<j}d\left(X_{i,t1},X_{j,t1}\right)$$

where *X*_*i,t*_ and *X*_*j,t*_ represent the community composition of replicate plots *i* and *j* in year *t*_1_, and *d* represents Bray–Curtis dissimilarity. For a treatment with six replicate plots, *R*_*t*1_ was calculated from all 15 pairwise dissimilarities among replicate plots in the same treatment and year. This represents the baseline heterogeneity (or “null state”) of the community at the start of the time interval.

The *deterministic component* of community compositional variation quantifies the directional shift in community structure. To quantify the deterministic component, we first calculated the mean compositional variation between the 2 years:

$$B_{t_{1},t_{2}}=\frac{1}{n^{2}}\sum_{i=1}^{n}\sum_{j=1}^{n}d\left(X_{i,t_{1}},X_{j,t_{2}}\right)$$

For a treatment with six replicate plots, (*B*_*t*1, *t*2_) was calculated from 36 cross‐year pairwise dissimilarities. The deterministic component of temporal community compositional change was then estimated as:

$$D_{t_{1},t_{2}}=B_{t_{1},t_{2}}-R_{t_{1}}$$

*Stochastic component* of temporal community compositional change quantifies the change in non‐directional dispersion (divergence or convergence) among replicates over time. In this study, Mantel tests showed that, for nearly all treatments, differences in soil physiochemical indices among replicate plots had no significant effect on either the plant or soil microbial compositional variation (Table S2). This preliminary evaluation suggested that the compositional variation among replicate plots could not be attributed to deterministic processes; hence, the stochastic processes should instead be the prevailing driver. To quantify the *stochastic component*, we first calculated the mean compositional variation of the year *t*_2_:

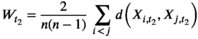




The stochastic component of temporal community compositional change was estimated as:

$$S_{t_{1},t_{2}}=W_{t_{2}}-R_{t_{1}}$$

Positive values of *S*_*t*1, *t*2_ indicate that among‐replicate dispersion increased through time, whereas negative values indicate that replicate communities became more similar to each other. Because Bray–Curtis dissimilarity ranges from 0 to 1, both *D*_*t*1,*t*2_ and *S*_*t*1,*t*2_ can theoretically range from −1 to 1. Positive values indicate an increase in the corresponding component relative to the initial reference state, whereas negative values indicate a decrease. The absolute value represents the magnitude of the deterministic or stochastic component.

For each treatment, *D*_*t*1,*t*2_ and *S*_*t*1,*t*2_ were calculated for all possible year pairs from 2010 to 2018. Similar to the calculation of the overall community succession rate using the time‐decay relationship, we fitted linear regressions between *D*_*t*1,*t*2_ or *S*_*t*1,*t*2_ and temporal distance *t*2–*t*1. The slope of each regression was used as the succession rate of the deterministic or stochastic component, representing the temporal rate of change in the corresponding component during community succession. The significance of each slope was assessed using a one‐sample *t*‐test between the original slope and bootstrapped slopes generated from random pairings of the original dataset with 999 permutations (Horner‐Devine et al. 2004; Zhou et al. 2008).

A null model framework was used to examine the results obtained from the experimentally‐based method. Beta (*β*) diversity, which represents the compositional variation between communities, is often used to infer plausible mechanisms of community assembly (such as the relative roles of deterministic versus stochastic processes). However, differing *β* diversity indexes may arise from a difference not only in the ecological processes but also in *α* as well as *γ* diversity. To exclude the influence of these other two diversity components, Chase (2010) developed a null model technique, which was dependent solely on species presence/absence data. In this study, to investigate, during succession, the relative contribution of deterministic and stochastic processes in driving the assembly of plant and soil microbial community, the community data of all plots of all treatments in all years was analysed together; in other words, the species or PLFA markers of all treatments across all years were taken as the species pool. In particular, following the steps outlined by Chase (2010) and Zhou et al. (2014), calculations were made to obtain these parameters: (1) observed species richness in each plot (e.g., *α*1 and *α*2 for plot 1 and 2, respectively) and the number of shared species (SSobs) between any two plots; (2) the total number of species detected in the ‘species pool’ (*γ* diversity) from all plots, and the proportion of plots occupied by each species; (3) the distribution of the expected shared species from null model (SSexp), by randomly drawing *α*1 and *α*2 species from the species pool, with the probability of a drawing species being proportional to its among‐plot occupancy. The SSexp and the expected Jaccard's similarity (Jexp) were obtained for each drawing, and the average Jaccard's similarity (Jexp) and its SD were then estimated based on 10,000 drawings (δexp). For any two plots, the relative magnitude of deterministic to stochastic processes for community assembly was quantified with the index of SES (standard effect size): SES = (Jobs−Jexp)/*δ*exp (Kraft et al. 2011; Zhou et al. 2014). Furthermore, the temporal turnover pattern in SES was analysed for each treatment and its slope was estimated to represent the succession rate.

### 2.5 Succession Rate of Soil Physicochemical Characteristics

For each treatment, temporal turnover in soil physicochemical characteristics was also evaluated using linear regression between Bray–Curtis dissimilarity and the time interval. The soil's available N content, pH, and water content were first standardized from 0 to 1, and these values were used to calculate the Bray–Curtis dissimilarity. The slope significance was also assessed by a one‐sample *t*‐test between the original slope and bootstrapped slopes from random pairings of the original set. That slope was a measure of the succession rate of soil physicochemical characteristics.

### 2.6 Statistical Analysis

Permutational multivariate analysis of variance (PERMANOVA) was performed using the adonis2 function in the R package vegan to test the effects of management strategy and N addition intensity on community composition based on Bray–Curtis dissimilarity. Restricted permutations were generated using the how() function in the R package permute, with permutations constrained within blocks and repeated observations from the same plot kept together. Non‐metric multidimensional scaling ordination (NMDS) based on the matrix of Bray–Curtis distances was conducted to reveal the yearly shifts (from 2010 through 2018) in both plant community composition and soil microbial biomarker composition under the nine different N addition intensities and two grassland management strategies (fencing vs. mowing). The effects of N deposition intensity and management strategy on soil available N content, pH, and water content were determined by two‐way repeated‐measures analysis of variance (ANOVA). The one‐sample *t*‐test was used to reveal whether fencing and mowing had disparate effects on these succession rates. Linear or quadratic regression was used to analyse the relationships between N deposition rate and succession rate. All statistical analyses were implemented in the R software v4.3.2 (R Core Team 2023) with the ‘vegan’ package unless indicated otherwise.

## 3 Results

Restricted PERMANOVA revealed that the management strategy, N addition intensity, and treatment time (year) had significant effects on both plant community composition and soil microbial biomarker composition (plant: *R* ^2^ = 0.043–0.082, *p* = 0.001; microbe: *R* ^2^ = 0.005–0.322, *p* = 0.001–0.043; Table S3). Consistently, the NMDS ordination maps showed that the successional patterns (from 2010 to 2018) of plant community composition and soil microbial biomarker composition differed under differing N addition intensities and management strategies (Figure S1A1–A18, B1–B18). Meanwhile, the composition of these communities exhibited significant patterns of temporal turnover (time‐decay relationships) for most N addition intensities under both strategies (*p* < 0.05; Figure S2A1–A18, B1–B18). Most interestingly, the succession rates of these communities displayed quadratic relationships with N addition intensity under either strategy (Figure 1a,c,d), albeit non‐significant for the plant community under mowing (Figure 1b).

**FIGURE 1:**
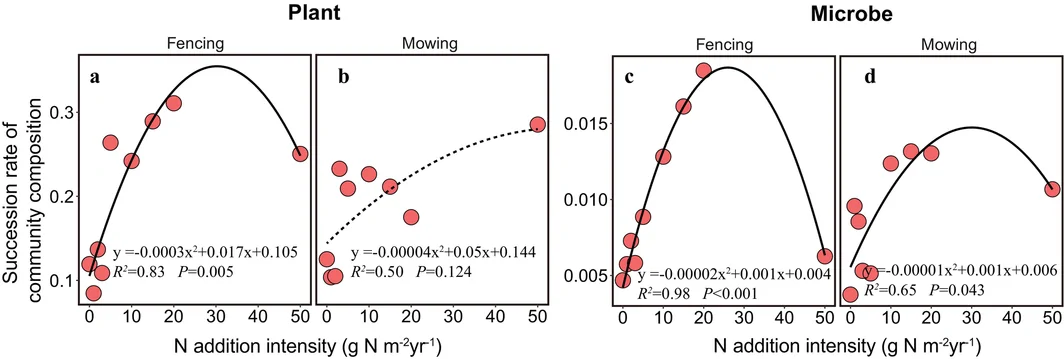
Effect of N addition intensity on the succession rate of plant (a, b) and soil microbial (c, d) community composition under fencing or mowing strategies.

Similar to the community composition exhibiting a pronounced pattern of temporal turnover (Figure S2A1–A18, B1–B18), their deterministic components showed significant patterns of temporal turnover for many N addition intensities under both strategies (*p* < 0.05; Figure S3A1–A18, B1–B18), and their stochastic components also exhibited similar significant patterns (*p* < 0.05; Figure S4A1–A18, B1–B18). Similar to the quadratic relationships between the succession rates of the community composition and N addition intensity (Figure 1), the succession rates of their deterministic community components also featured significant quadratic relationships with N addition intensity (*p* < 0.05; Figure 2a–d). In contrast, the succession rate of the stochastic component of the plant community under mowing (Figure 2f), and likewise that of the microbial community under fencing (Figure 2g), showed significant linear relationships with N addition intensity (*p* < 0.05). However, the succession rate of the stochastic component of the plant community under fencing (Figure 2e) and that of the microbial community under mowing (Figure 2h) did not have any linear or quadratic relationships with N addition rate. Further, the SES values of both plant and microbial communities also were distinguished by significant patterns of temporal turnover for many N addition intensities under both strategies (*p* < 0.05; Figure S5A1–A18, B1–B18), and their succession rates also had marginally significant quadratic relationships with N addition intensity (*p* < 0.10; Figure S6a–d).

**FIGURE 2:**
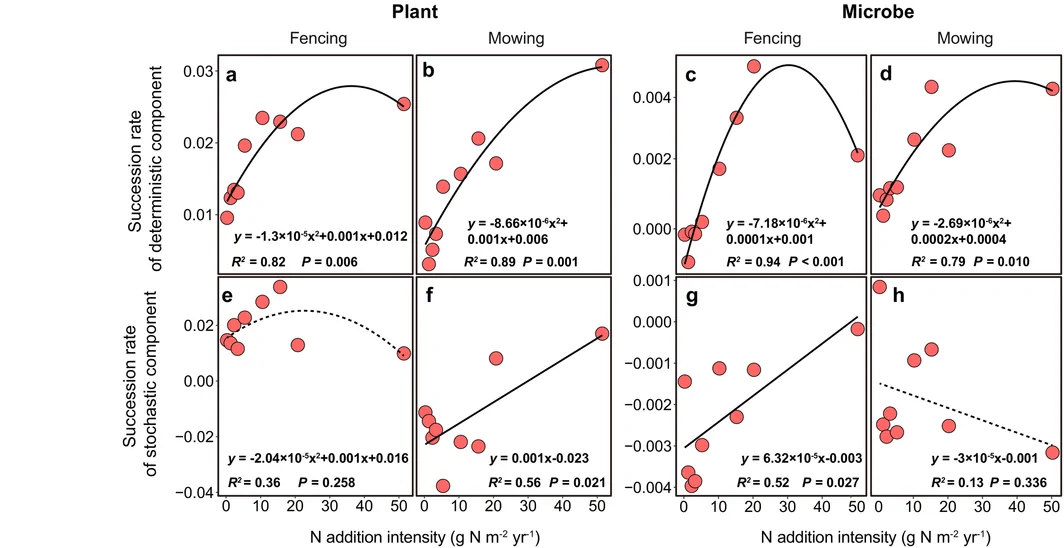
Effect of N addition intensity on the succession rate of the deterministic (a–d) or stochastic (e–h) community component of plants and soil microbes under fencing or mowing.

Two‐way repeated‐measures ANOVA revealed that while N addition significantly increased the soil's available N content, mowing further elevated that content relative to fencing (*p* < 0.05; Figure S7a). Meanwhile, N addition significantly lowered the soil pH (*p* < 0.05; Figure S7b), while mowing significantly decreased the soil's water content relative to fencing (*p* < 0.05; Figure S7c). Similar to the community composition exhibiting a significant pattern of temporal turnover (Figure S2), soil physicochemical characteristics (integrating soil pH, available N and water content) also exhibited significant patterns of temporal turnover for all N addition intensities under both strategies (*p* < 0.05; Figure S8). Like the quadratic relationships between the succession rates of the community composition and N addition intensity (Figure 1), the succession rate of soil physicochemical characteristics also had significant quadratic relationships with N addition intensity under either strategy (*p* < 0.05; Figure 3a,b).

**FIGURE 3:**
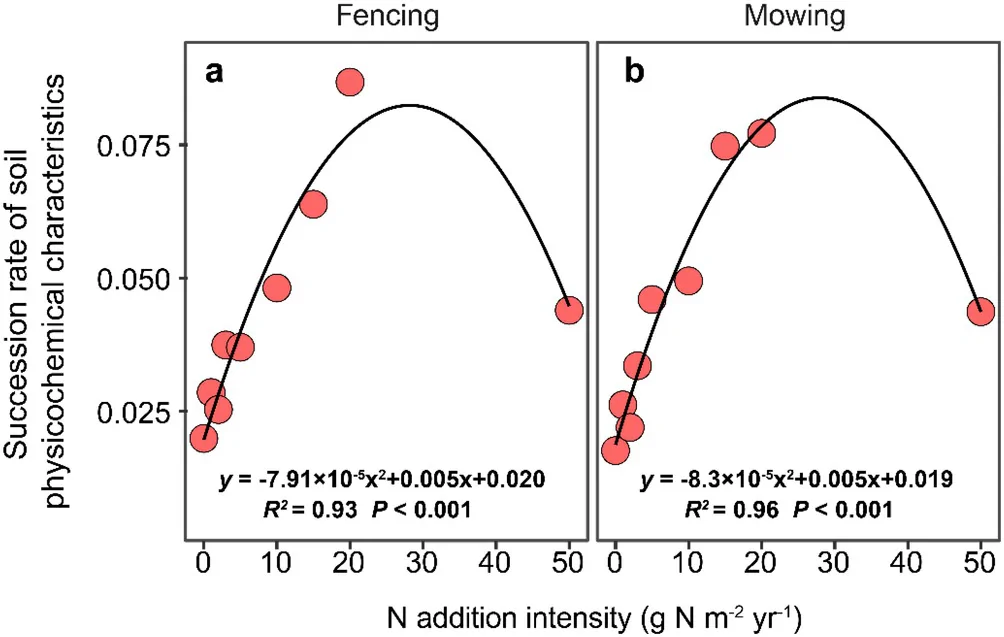
Effect of N addition intensity on the succession rate of soil physicochemical characteristics (integrating soil pH, available N content, and water content) under fencing (a) or mowing (b) strategies.

There were significant correlations between the succession rate of soil physicochemical characteristics and that of community composition for plants under fencing (*p* < 0.05; Figure 4a) and for soil microbes under fencing as well as mowing (Figure 4c,d), although not so for the plants under mowing (*p* > 0.05; Figure 4b). Consistently, significant correlations were found between the succession rate of soil physicochemical characteristics and that of the deterministic component for plants under fencing (*p* < 0.05; Figure 4e) and for soil microbes under both fencing and mowing (Figure 4g,h), yet not for plants under mowing (*p* > 0.05; Figure 4f). In contrast, there were non‐significant correlations between the succession rate of soil physicochemical characteristics and that of the stochastic component for these communities under both strategies (*p* > 0.05; Figure 4i–l).

**FIGURE 4:**
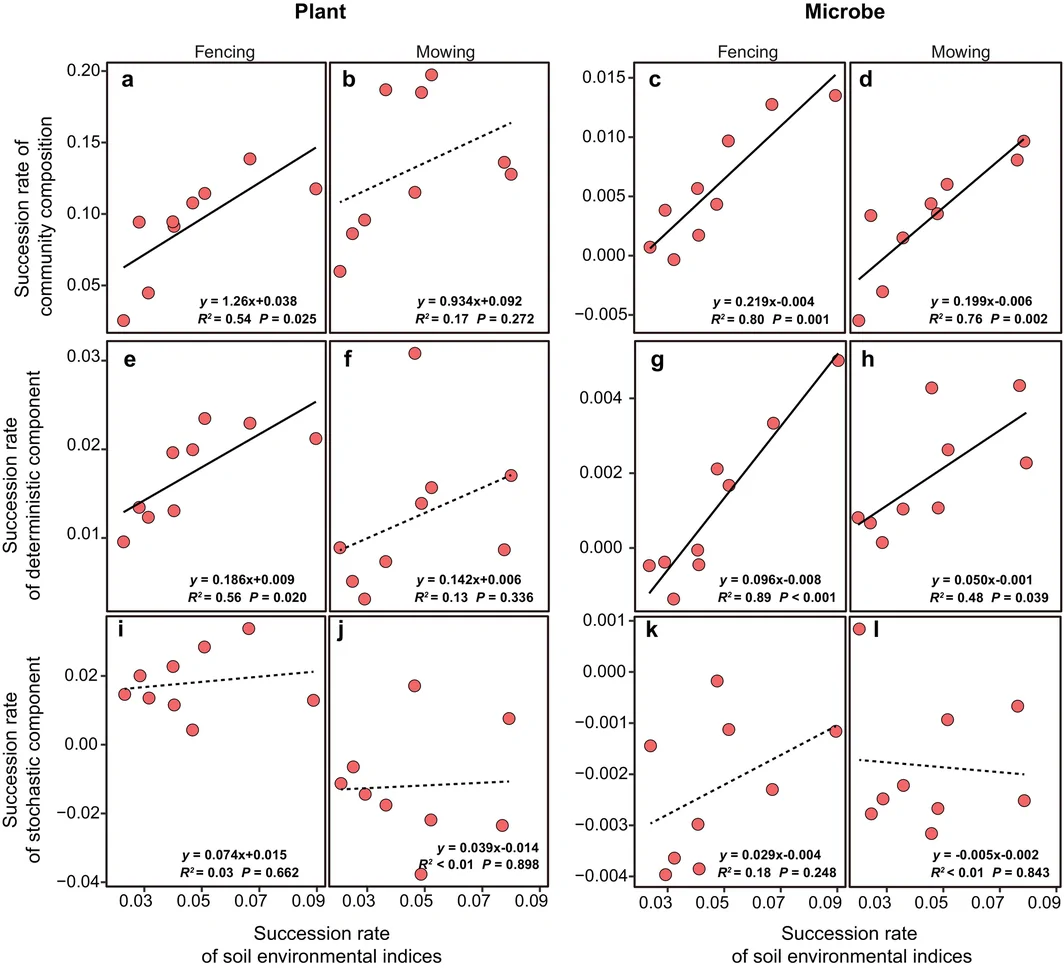
Linear relationships between the succession rate of soil physicochemical characteristics and that of community composition (a–d), and of the deterministic (e–h) or stochastic (i–l) community component of plants and soil microbes, under fencing or mowing strategies.

Across the N addition intensities of 0 to 20 g N m^−2^ yr^−1^, one‐sample *t*‐tests revealed that the succession rates of plant community composition were significantly smaller under fencing than mowing (*p* < 0.05; Figure 5a). However, the succession rates were significantly larger under fencing than mowing for the plant community's deterministic component and also its stochastic component (Figure 5a). Meanwhile, the succession rates of soil microbial biomarker composition were significantly larger under fencing than mowing (*p* < 0.05; Figure 5b), while the succession rates of the deterministic microbial community component under fencing and those under mowing had a non‐significant difference. Conversely, the succession rates of the stochastic microbial community component were significantly smaller under fencing than mowing (Figure 5b).

**FIGURE 5:**
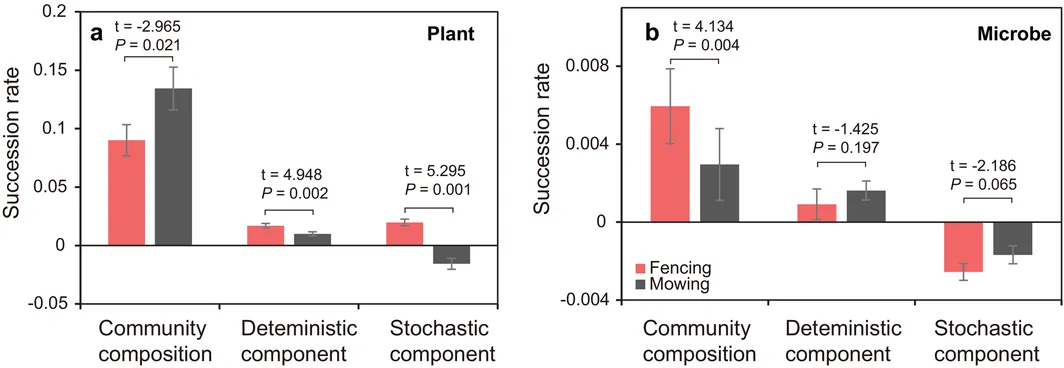
Effect of grassland management strategy (fencing vs. mowing) on the succession rate of community composition and that of the stochastic or deterministic community component of plants (a) and soil microbes (b). The bars represent one standard error.

## 4 Discussion

Examining biological adaptation at the community composition level to global change is a central but still unresolved goal in community ecology (Travisano et al. 1995). Here we tried to answer this question by considering the relationship between global change intensity and the community succession rate, a critical index that could quantify the rate of community compositional variation across time (Guo et al. 2018; Shimadzu et al. 2015). We hypothesized that when environmental change can be accommodated through compositional adjustment, succession rates should increase with rising stress up to a threshold, reflecting accelerated species replacement driven by processes such as intensified species competition, species loss, and colonization under new conditions (Bai et al. 2015; Xu et al. 2012; Zhang, Stevens, et al. 2016). Beyond this threshold, further compositional adjustment becomes constrained, leading to a plateau or decline in succession rates, indicating adaptation failure. Our results support this framework: succession rates of both plant and microbial communities increased from 0 to 20 g N m^−2^ yr^−1^, indicating effective compositional adaptation, but decreased sharply at 50 g N m^−2^ yr^−1^, signalling a breakdown of adaptive capacity. This unimodal response aligns with findings that nitrogen enrichment generally accelerates community turnover in the short to medium term (Inouye and Tilman 1995; Knelman et al. 2014) but may suppress succession under chronic, high‐intensity loading (Liang et al. 2020). The decline in succession rate at the highest intensity can be attributed to the exhaustion of the native species pool. Over the course of its long geological history, the soil of the vast Eurasian steppe has been generally deficient in N and is often neutral or weakly basic (Yuan et al. 2006). In the species pool of this broad steppe area, very few species have experienced and adapted to the excessive N and acidified soil caused by the highest N intensity (Bai et al. 2010; Zhao et al. 2022). Consequently, the community rapidly stabilizes into a depauperate state with minimal further species gain or loss, resulting in low temporal turnover.

Although both deterministic and stochastic ecological processes can drive community species dynamics (Chase 2010; Nemergut et al. 2013; Zhou and Ning 2017), stochastic ones can even play more important roles than deterministic ones in many cases (Wang et al. 2024; Bick et al. 2025; Li et al. 2022). Nevertheless, our experimental results demonstrate that deterministic rather than stochastic processes were the prevailing mechanism underpinning adaptation of community composition to global changes. Firstly, while the succession rate of the deterministic community components shows quadratic relationships with N addition intensity, mirroring the pattern of overall community succession, the stochastic community components do not. Secondly, the succession rate of soil physicochemical characteristics also has quadratic relationships with N addition intensity, like that for the entire community composition. Crucially, the succession rate of soil physicochemical characteristics is significantly correlated with the succession rate of the entire community composition as well as that of the deterministic community component, but is not correlated with that of the stochastic community component. This suggests that N addition alters the soil environment in a way that imposes a strong, directional filter on species survival and performance—selecting for taxa tolerant to low pH, high N availability, and possibly aluminium toxicity (Chen et al. 2013; Hu et al. 2024; Yang et al. 2020)—rather than merely amplifying random demographic or dispersal events. Actually, the trend of first increasing then decreasing for the succession rate of soil physicochemical characteristics in response to a rising N addition intensity suggests the highest N addition intensity was so great that soil physicochemical indices could not buffer changes caused by it anymore. Finally, the quadratic relationships between the SES succession rate from the null model method and N addition intensity further supported the results obtained from the experimentally based method. Taken together, N addition evidently alters the soil physicochemical indices, which deterministically select for certain species—rather than promoting or restraining the stochastic birth/death and migration/colonization of species—thus resulting in the adaptation process. Since we only quantified the three key soil physicochemical indices of pH, available N content, and water content during this long‐term survey, the succession and effect of many other soil indices should also be monitored in future similar studies.

The results also demonstrated that management strategies could affect the rate of succession and adaptation of biological communities to global changes by mediating stochastic ecological processes. Actually, in investigating the influence of different management strategies on the adaptability of biological communities to N addition, we focused only on the relatively low N addition intensities (0–20 g N m^−2^ yr^−1^), since adaptive compositional adjustment was constrained at the highest intensity of 50 g N m^−2^ yr^−1^. In particular, and logically consistent with the higher succession rates of plant community composition under mowing than fencing, the succession rate of the plant community's stochastic component was lower under mowing than fencing, being actually < 0 under mowing. This is because the mowing treatment removed aboveground plants annually and hindered stochastic colonization by plant species (Zhang et al. 2017, 2025). Meanwhile, being logically consistent with the higher succession rates of soil microbial biomarker composition under fencing than mowing, the succession rates of the microbial community's stochastic component were lower under fencing than mowing, actually also < 0 under fencing. One possible explanation is that fencing, by excluding grazing‐related disturbances and promoting aboveground biomass and litter accumulation, may alter microbial dispersal and colonization pathways, thereby reducing the stochastic component of soil microbial compositional change (Liu et al. 2025; Zhou et al. 2025). Altogether, the processes of stochastic birth/death and migration/colonization could slow down the directional adaptive process, and thus, management strategies that could curtail these stochastic processes would promote the deterministic adaptation process of biological communities to global changes.

Overall, from the perspective of the relation between the community succession rate and global change intensity, we successfully inferred whether adaptation of biological communities occurred under a certain N addition intensity through changes in species composition. We went on to identify the deterministic adaptive mechanism as being responsible, and also examined how management strategies affect this adaptive process. Our analytical approach should be helpful to investigate the adaptive response and mechanisms of various biological communities to other types of global changes under different management strategies in future studies. Nonetheless, the plant and soil microbial communities did show similar adaptive responses to N addition intensity. If more advanced technologies, such as meta‐transcriptomic sequencing rather than the low‐resolution PLFA method, are utilized to measure the active community composition of soil microorganisms, divergent adaptive responses between plant and soil microbial communities may be observed, which warrants careful examination in future studies. Furthermore, the relative contributions of physicochemical, compositional, and genetic adaptations to global changes need to be rigorously investigated in future research. Still, according to our experimental results, to facilitate adaptation at the community compositional level through species composition change, global change intensities should be maintained below certain adaptive thresholds, together with the appropriate implementation of reasonable deterministic (e.g., restoring soil environment) and/or stochastic (e.g., adding seeds of adaptive species) strategies.

## Author Contributions

**Tingting Li:** data curation, investigation, formal analysis, visualization, project administration, writing – original draft, writing – review and editing. **Rong Mao:** writing – review and editing, investigation, methodology, project administration. **Yang Zhang:** data curation, writing – review and editing. **Ximei Zhang:** conceptualization, writing – original draft, funding acquisition, project administration, resources, writing – review and editing.

## Funding

This work was supported by the Double Thousand Plan of Jiangxi Province (jxsq2023102216), the National Natural Science Foundation of China (U21A20188, 32071547), the Young Top‐Notch Talent Support Program of the National High‐level Talents Special Support Plan and the Agricultural Science and Technology Innovation Program.

## Conflicts of Interest

The authors declare no conflicts of interest.

## Supporting information


**Figure S1:** Succession patterns (from 2010 through 2018) of plant (A1–A18) and microbial (B1–B18) community composition under different N addition intensities and grassland management strategies, as revealed by non‐metric multidimensional scaling ordination (NMDS) basing on Bray–Curtis distances. Community compositional data of all treatments were combined for the NMDS analysis, and then the NMDS axes 1 and 2 were separated for each treatment to draw the figure. N0 to N50 represent the N addition intensities of 0 to 50 g N m^−2^ yr^−1^, respectively.
**Figure S2:** Time‐decay Relationships (TDRs) of plant (A1–A18) and soil microbial communities (B1–B18) under different N addition intensities and two grassland management strategies.
**Figure S3:** Temporal turnover of the deterministic component of plant (A1–A18) and soil microbial (B1–B18) community composition under fencing or mowing strategies.
**Figure S4:** Temporal turnover of the stochastic component of plant (A1–A18) and soil microbial (B1–B18) community composition under fencing or mowing strategies.
**Figure S5:** Temporal turnover of SES of plant (A1–A18) and soil microbes (B1–B18) under fencing or mowing strategies.
**Figure S6:** Effect of N addition intensity on the succession rate of the SES of plants and soil microbes under fencing or mowing strategies.
**Figure S7:** Effect of N addition intensity on soil's available N content (a), pH (b), and water content (c).
**Figure S8:** Temporal turnover of soil physicochemical characteristics (integrating soil pH, available N content, and water content) under different N addition intensities and two grassland management strategies.
**Table S1:** The corresponding microbial groups for PLFA markers.
**Table S2:** Effect (*p*‐value) of soil physicochemical indices among replicate plots on plant or soil microbial community composition for each treatment of each year revealed by Mantel test.
**Table S3:** Effect of management strategy, N addition intensity, year, and their interaction on the compositional variations of plant and soil microbial communities revealed by restricted permutational multivariate analysis of variance (Restricted PERMANOVA).

## Data Availability

The data that support the findings of this study are available on request from the corresponding author. The data are not publicly available due to privacy or ethical restrictions.
