# Supplementary material for: Inferring Adaptation at Community Composition Level From the Relationship Between Succession Rate and Global Change Intensity
Source: Environ Microbiol Rep. 2026 Sep 30;18(5):e70426. doi: 10.1111/1758-2229.70426 (PMC13626865; doi:10.1111/1758-2229.70426)
Supplement: Supplementary file 1 [file EMI4-18-e70426-s001.docx]

**Fig. S1**. Succession patterns (from 2010 through 2018) of plant (A1-A18) and microbial (B1-B18) community composition under different N addition intensities and grassland management strategies, as revealed by non-metric multidimensional scaling ordination (NMDS) basing on Bray–Curtis distances. Community compositional data of all treatments were combined for the NMDS analysis, and then the NMDS axes 1 and 2 were separated for each treatment to draw the figure. N0 to N50 represent the N addition intensities of 0 to 50 g N m^-2^ yr^-1^, respectively.

**Fig. S2**. Time-decay Relationships (TDRs) of plant (A1–A18) and soil microbial communities (B1–B18) under different N addition intensities and two grassland management strategies.

**Fig. S3**. Temporal turnover of the deterministic component of plant (A1–A18) and soil microbial (B1–B18) community composition under fencing or mowing strategies.

**Fig. S4**. Temporal turnover of the stochastic component of plant (A1–A18) and soil microbial (B1–B18) community composition under fencing or mowing strategies.

**Fig. S5**. Temporal turnover of SES of plant (A1–A18) and soil microbes (B1–B18) under fencing or mowing strategies.

## Fig. S6. Effect of N addition intensity on the succession rate of the SES of plants and soil microbes under fencing or mowing strategies.

## Fig. S7. Effect of N addition intensity on soil’s available N content (a), pH (b), and water content (c).

**Fig. S8**. Temporal turnover of soil physicochemical characteristics (integrating soil pH, available N content, and water content) under different N addition intensities and two grassland management strategies.

## Table S1. The corresponding microbial groups for PLFA markers.

| PLFA marker | Microbial group |
| --- | --- |
| 14:00, 15:00, 16:00, 17:00, 18:00, 20:00 | General bacterial |
| 16:1 w9c, 17:1 w8c, 18:1 w5c, 20:1 w9c, cy17:0 | Gram negative bacteria |
| a15:0, a17:0, a17:1w9c, i14:0, i15:0, i16:0, i17:0 | Gram positive bacteria |
| 18:1 w9c | Fungi |
| 16:1 w5c | Arbuscular Mycorrhizae |

## Table S2. Effect (*P* value) of soil physicochemical indices among replicate plots on plant or soil microbial community composition for each treatment of each year revealed by Mantel test.

| Indices | Community-treatment | Year | N0 | N1 | N2 | N3 | N5 | N10 | N15 | N20 | N50 |
| --- | --- | --- | --- | --- | --- | --- | --- | --- | --- | --- | --- |
| Available nitrogen content | Plant-fencing | 2010 | 0.11 | 0.51 | 0.93 | 0.68 | 0.89 | 0.63 | 0.25 | 0.85 | 0.33 |
|  |  | 2011 | 0.22 | 0.31 | 0.44 | 0.25 | 0.30 | 0.93 | 0.46 | 0.90 | 0.70 |
|  |  | 2012 | 0.83 | 0.20 | **0.01** | 0.57 | 0.19 | 0.75 | 0.59 | **0.05** | 0.87 |
|  |  | 2013 | 0.35 | 0.44 | 0.31 | 0.35 | 0.65 | 0.08 | 0.48 | 0.27 | 0.23 |
|  |  | 2014 | 0.37 | 0.06 | 0.66 | 0.72 | 0.51 | 0.97 | 0.68 | 0.51 | 0.58 |
|  |  | 2015 | 0.67 | 0.30 | 0.88 | 0.98 | 0.26 | 0.81 | 0.99 | 0.68 | 0.38 |
|  |  | 2016 | 0.50 | 0.96 | 0.38 | 0.36 | 0.36 | 0.58 | 0.63 | 0.68 | 0.08 |
|  |  | 2017 | 0.08 | 0.33 | 0.59 | 0.84 | 0.13 | 0.46 | 0.96 | 0.99 | 0.15 |
|  |  | 2018 | 0.56 | 0.50 | 0.59 | 0.81 | 0.78 | 0.22 | 0.37 | 0.27 | 0.23 |
|  | Plant-mowing | 2010 | 0.52 | 0.44 | 0.59 | 0.16 | 0.62 | 0.89 | 0.31 | 0.10 | 0.17 |
|  |  | 2011 | 0.78 | **0.05** | 0.55 | 0.20 | 0.84 | 0.35 | 0.16 | 0.52 | 0.20 |
|  |  | 2012 | 0.09 | 0.11 | **0.04** | 0.47 | 0.20 | **0.03** | 0.55 | 0.48 | 0.37 |
|  |  | 2013 | 0.22 | 0.89 | 0.93 | 0.76 | 0.30 | 0.52 | 0.67 | **0.04** | 0.49 |
|  |  | 2014 | 0.54 | 0.80 | 0.31 | 0.31 | 0.18 | 0.44 | 0.79 | 0.94 | 0.44 |
|  |  | 2015 | 0.46 | 0.76 | 0.27 | 0.80 | 0.65 | 0.44 | 0.64 | 0.66 | **0.04** |
|  |  | 2016 | 0.09 | 0.33 | 0.08 | 0.77 | 0.58 | 0.73 | 0.40 | 0.09 | 0.58 |
|  |  | 2017 | 0.83 | 0.73 | 0.30 | 0.30 | 0.63 | **0.03** | 0.66 | 0.15 | 0.33 |
|  |  | 2018 | 0.36 | 0.14 | 0.36 | 0.28 | 0.1 | 0.07 | **0.01** | **0.03** | 0.14 |
|  | Microbe-fencing | 2010 | 0.22 | 0.07 | 0.99 | 0.93 | 0.11 | 0.96 | 0.14 | 0.70 | 0.48 |
|  |  | 2011 | 0.13 | 0.63 | 0.80 | 0.35 | 0.08 | 0.68 | 0.08 | 0.39 | 0.84 |
|  |  | 2012 | 0.76 | 0.68 | 0.54 | 0.70 | 0.53 | 0.32 | 0.69 | 0.58 | 0.16 |
|  |  | 2013 | 0.73 | 0.59 | 0.64 | 0.34 | 0.88 | 0.96 | 0.07 | 0.75 | 0.69 |
|  |  | 2014 | 0.40 | 0.54 | 0.24 | 0.61 | 0.49 | 0.73 | 0.40 | 0.56 | 0.83 |
|  |  | 2015 | 0.96 | 0.75 | 0.14 | 0.09 | 0.89 | 0.61 | 0.76 | 0.74 | **0.02** |
|  |  | 2016 | 0.08 | 0.12 | 0.57 | 0.18 | 0.89 | 0.93 | 0.14 | 0.07 | 0.68 |
|  |  | 2017 | 0.09 | 0.89 | 0.93 | 0.60 | 0.91 | 0.67 | 0.48 | 0.65 | 0.41 |
|  |  | 2018 | 0.32 | 0.41 | 0.55 | 0.92 | 0.31 | 0.61 | 0.40 | 0.94 | 0.29 |
|  | Microbe-mowing | 2010 | 0.25 | 0.39 | 0.73 | 0.22 | 0.76 | 0.67 | 0.58 | 0.94 | 0.14 |
|  |  | 2011 | 0.23 | 0.67 | 0.76 | 0.56 | 0.54 | 0.40 | 0.42 | 0.28 | 0.75 |
|  |  | 2012 | 0.92 | 0.79 | 0.93 | 0.21 | 0.53 | **0.05** | 0.54 | 0.12 | 0.67 |
|  |  | 2013 | **0.02** | 0.42 | 0.25 | 0.74 | 0.99 | 0.29 | 0.38 | 0.96 | 0.06 |
|  |  | 2014 | 0.49 | 0.45 | 0.80 | 0.56 | 0.59 | 0.11 | 0.53 | **0.04** | 0.83 |
|  |  | 2015 | 0.71 | **0.01** | 0.19 | 0.10 | 0.39 | 0.90 | 0.62 | 0.94 | 0.83 |
|  |  | 2016 | 0.89 | 0.40 | 0.46 | 0.84 | 0.77 | 0.19 | 0.46 | 0.46 | 0.06 |
|  |  | 2017 | 0.98 | 0.03 | 0.46 | 0.34 | 0.20 | 0.75 | 0.55 | 0.54 | **0.05** |
|  |  | 2018 | 0.29 | 0.65 | 0.09 | 0.08 | 0.49 | 0.13 | 0.25 | 0.18 | 0.63 |

| Indices | Community-treatment | Year | N0 | N1 | N2 | N3 | N5 | N10 | N15 | N20 | N50 |
| --- | --- | --- | --- | --- | --- | --- | --- | --- | --- | --- | --- |
| Soil pH | Plant-fencing | 2010 | 0.21 | 0.47 | 0.41 | 0.33 | 0.70 | 0.37 | 0.22 | 0.16 | 0.92 |
|  |  | 2011 | 0.53 | 0.91 | 0.25 | 0.21 | 0.77 | 0.87 | 0.21 | 0.21 | 0.36 |
|  |  | 2012 | 0.64 | 0.72 | 0.13 | 0.17 | 0.89 | 0.34 | 0.62 | 0.15 | 0.47 |
|  |  | 2013 | 0.67 | 0.55 | **0.04** | 0.36 | 0.98 | 0.66 | 0.12 | 0.16 | 0.23 |
|  |  | 2014 | 0.15 | 0.49 | 0.41 | 0.42 | 0.61 | 0.31 | 0.78 | **0.05** | 0.68 |
|  |  | 2015 | 0.54 | 0.19 | 0.13 | 0.39 | 0.89 | 0.33 | 0.14 | 0.68 | **0.01** |
|  |  | 2016 | 0.84 | 0.70 | 0.78 | **0.01** | 0.34 | 0.38 | 0.36 | 0.56 | 0.47 |
|  |  | 2017 | 0.72 | 0.26 | **0.02** | 0.93 | 0.68 | 0.77 | 0.54 | **0.01** | 0.22 |
|  |  | 2018 | 0.67 | 0.88 | 0.34 | 0.20 | 0.91 | 0.09 | 0.78 | 0.91 | 0.54 |
|  | Plant-mowing | 2010 | 0.80 | 0.31 | 0.06 | 0.13 | 0.07 | 0.58 | 0.42 | 0.98 | 0.36 |
|  |  | 2011 | 0.31 | 0.86 | 0.37 | 0.58 | 0.66 | 0.12 | 0.74 | 0.59 | 0.44 |
|  |  | 2012 | 0.70 | 0.47 | **0.02** | 0.99 | 0.99 | 0.09 | **0.05** | 0.98 | 0.46 |
|  |  | 2013 | 0.97 | 0.15 | **0.05** | 0.92 | 0.38 | 0.27 | 0.16 | 0.19 | 0.51 |
|  |  | 2014 | 0.75 | 0.8 | 0.07 | 0.94 | 0.14 | 0.46 | 0.91 | 0.32 | 0.23 |
|  |  | 2015 | **0.02** | 0.31 | **0.99** | 0.63 | 0.16 | 0.59 | 0.59 | 0.48 | 0.56 |
|  |  | 2016 | 0.41 | 0.41 | 0.24 | 0.41 | 0.50 | 0.49 | 0.07 | 0.74 | 0.88 |
|  |  | 2017 | 0.70 | 0.62 | 0.45 | 0.33 | 0.66 | 0.61 | 0.99 | 0.30 | 0.56 |
|  |  | 2018 | 0.30 | 0.95 | **0.01** | 0.53 | 0.24 | 0.19 | 0.36 | 0.08 | 0.40 |
|  | Microbe-fencing | 2010 | **0.03** | 0.24 | 0.23 | 0.79 | 0.95 | 0.50 | 0.46 | 0.10 | 0.11 |
|  |  | 2011 | 0.25 | 0.17 | **0.01** | 0.28 | 0.49 | 0.37 | 0.16 | 0.60 | 0.21 |
|  |  | 2012 | 0.73 | 0.57 | 0.81 | 0.77 | 0.54 | 0.89 | 0.12 | 0.56 | 0.23 |
|  |  | 2013 | 0.92 | 0.41 | **0.04** | 0.37 | 0.60 | 0.14 | 0.82 | **0.04** | 0.26 |
|  |  | 2014 | 0.54 | 0.38 | 0.08 | 0.91 | 0.50 | 0.36 | 0.89 | 0.08 | 0.66 |
|  |  | 2015 | 0.91 | 0.25 | 0.47 | 0.07 | 0.39 | 0.87 | 0.09 | 0.25 | 0.59 |
|  |  | 2016 | 0.87 | 0.94 | **0.04** | **0.03** | 0.81 | 0.42 | 0.08 | 0.83 | 0.8 |
|  |  | 2017 | 0.26 | 0.78 | 0.26 | 0.51 | 0.34 | **0.02** | 0.42 | **0.05** | 0.59 |
|  |  | 2018 | 0.81 | **0.03** | 0.17 | 0.18 | 0.18 | 0.55 | **0.05** | 0.20 | 0.82 |
|  | Microbe-mowing | 2010 | 0.35 | 0.08 | 0.48 | 0.38 | 0.15 | 0.46 | 0.12 | 0.40 | 0.08 |
|  |  | 2011 | **0.01** | 0.09 | 0.62 | 0.36 | 0.11 | 0.50 | 0.35 | **0.03** | 0.38 |
|  |  | 2012 | 0.31 | 0.63 | 0.55 | 0.64 | 0.55 | 0.75 | 0.44 | 0.66 | 0.07 |
|  |  | 2013 | 0.86 | 0.78 | 0.12 | 0.90 | 0.37 | 0.07 | 0.60 | 0.44 | 0.45 |
|  |  | 2014 | 0.77 | 0.12 | 0.97 | 0.62 | 0.58 | 0.14 | 0.61 | 0.93 | 0.42 |
|  |  | 2015 | 0.82 | 0.69 | 0.78 | 0.11 | 0.50 | 0.71 | 0.24 | 0.29 | 0.23 |
|  |  | 2016 | 0.11 | 0.83 | 0.54 | 0.29 | 0.73 | **0.01** | 0.80 | 0.78 | 0.40 |
|  |  | 2017 | 0.98 | 0.79 | 0.48 | 0.25 | 0.47 | 0.13 | 0.42 | 0.16 | **0.05** |
|  |  | 2018 | 0.54 | 0.89 | 0.23 | 0.91 | 0.40 | 0.33 | 0.16 | 0.62 | 0.48 |

| Indices | Community-treatment | Year | N0 | N1 | N2 | N3 | N5 | N10 | N15 | N20 | N50 |
| --- | --- | --- | --- | --- | --- | --- | --- | --- | --- | --- | --- |
| Soil water content | Plant-fencing | 2010 | 0.24 | **0.05** | 0.08 | 0.07 | 0.29 | 0.70 | 0.82 | 0.11 | 0.06 |
|  |  | 2011 | 0.25 | 0.77 | 0.44 | 0.29 | 0.62 | 0.85 | 0.59 | 0.08 | 0.74 |
|  |  | 2012 | 0.55 | 0.72 | 0.91 | 0.57 | 0.16 | 0.23 | 0.96 | 0.84 | 0.07 |
|  |  | 2013 | **0.02** | 0.6 | 0.61 | 0.13 | 0.21 | 0.26 | 0.73 | 0.87 | 0.10 |
|  |  | 2014 | **0.01** | 0.25 | 0.40 | 0.06 | 0.13 | 0.36 | 0.44 | 0.66 | 0.32 |
|  |  | 2015 | 0.36 | 0.87 | 0.44 | 0.82 | 0.35 | 0.77 | 0.60 | 0.52 | 0.80 |
|  |  | 2016 | 0.59 | 0.20 | 0.72 | 0.63 | 0.69 | 0.73 | 0.86 | 0.28 | 0.31 |
|  |  | 2017 | 0.06 | 0.80 | 0.79 | 0.60 | 0.45 | 0.75 | 0.32 | 0.35 | 0.42 |
|  |  | 2018 | 0.92 | 0.98 | 0.23 | 0.15 | 0.28 | 0.59 | 0.13 | 0.92 | 0.40 |
|  | Plant-mowing | 2010 | 0.89 | 0.45 | 0.73 | 0.91 | 0.33 | 0.15 | 0.16 | 0.25 | 0.56 |
|  |  | 2011 | 0.12 | 0.43 | 0.24 | 0.84 | 0.67 | 0.52 | 0.86 | 0.63 | 0.90 |
|  |  | 2012 | 0.11 | **0.04** | 0.10 | 0.78 | 0.90 | 0.14 | 0.61 | 0.37 | 0.08 |
|  |  | 2013 | 0.12 | 0.49 | 0.76 | 0.34 | 0.63 | 0.89 | 0.90 | 0.54 | 0.70 |
|  |  | 2014 | 0.48 | 0.42 | 0.72 | 0.40 | 0.62 | 0.45 | 0.78 | 0.16 | **0.05** |
|  |  | 2015 | 0.67 | 0.63 | **0.05** | **0.05** | 0.21 | 0.13 | 0.11 | 0.95 | 0.91 |
|  |  | 2016 | 0.43 | 0.73 | 0.43 | 0.47 | 0.25 | 0.71 | 0.25 | 0.11 | 0.36 |
|  |  | 2017 | 0.97 | 0.68 | 0.23 | 0.93 | 0.83 | 0.13 | 0.64 | 0.32 | 0.91 |
|  |  | 2018 | 0.15 | 0.37 | **0.03** | 0.88 | 0.54 | 0.28 | 0.81 | 0.14 | 0.81 |
|  | Microbe-fencing | 2010 | 0.47 | 0.15 | 0.13 | 0.19 | 0.92 | 0.91 | 0.80 | 0.18 | 0.87 |
|  |  | 2011 | 0.09 | 0.75 | 0.73 | 0.07 | 0.45 | 0.60 | 0.83 | 0.79 | 0.49 |
|  |  | 2012 | 0.26 | 0.53 | 0.43 | 0.20 | 0.51 | 0.73 | 0.10 | 0.87 | 0.14 |
|  |  | 2013 | 0.18 | 0.50 | 0.54 | 0.95 | 0.13 | 0.68 | 0.51 | 0.73 | 0.89 |
|  |  | 2014 | **0.01** | 0.68 | 0.98 | 0.58 | 0.60 | 0.81 | 0.27 | 0.57 | 0.59 |
|  |  | 2015 | 0.80 | 0.70 | 0.43 | 0.87 | 0.40 | 0.40 | **0.05** | 0.48 | 0.55 |
|  |  | 2016 | 0.47 | 0.99 | 0.17 | 0.29 | 0.65 | **0.04** | 0.43 | 0.91 | 0.84 |
|  |  | 2017 | 0.13 | 0.64 | 0.29 | 0.27 | 0.61 | 0.07 | 0.64 | 0.55 | 0.12 |
|  |  | 2018 | 0.66 | 0.08 | 0.11 | 0.07 | 0.43 | 0.46 | 0.82 | 0.45 | 0.24 |
|  | Microbe-mowing | 2010 | 0.37 | 0.98 | 0.28 | 0.64 | 0.25 | **0.04** | 0.30 | 0.83 | 0.53 |
|  |  | 2011 | 0.18 | 0.39 | 0.80 | 0.78 | 0.82 | 0.82 | 0.36 | **0.02** | 0.16 |
|  |  | 2012 | 0.91 | 0.84 | 0.51 | 0.81 | **0.04** | 0.46 | 0.14 | 0.35 | **0.03** |
|  |  | 2013 | 0.21 | 0.65 | 0.58 | 0.86 | 0.19 | 0.47 | 0.76 | 0.62 | 0.45 |
|  |  | 2014 | 0.17 | 0.50 | 0.15 | 0.90 | 0.90 | 0.75 | 0.57 | 0.44 | 0.39 |
|  |  | 2015 | 0.77 | 0.99 | 0.98 | 0.57 | 0.97 | 0.75 | 0.65 | 0.07 | 0.88 |
|  |  | 2016 | 0.70 | 0.74 | 0.38 | 0.14 | 0.30 | 0.46 | 0.56 | 0.95 | 0.64 |
|  |  | 2017 | 0.95 | 0.48 | 0.24 | 0.67 | 0.21 | 0.53 | 0.33 | 0.73 | 0.79 |
|  |  | 2018 | 0.69 | 0.20 | **0.05** | 0.17 | 0.45 | 0.56 | 0.86 | 0.23 | 0.55 |

Significant *P* values (< 0.05) are bolded. N0-N50 represent nitrogen addition intensities of 0-50 g N m^-2^ yr^-1^, respectively.

## Table S3. Effect of management strategy, N addition intensity, year, and their interaction on the compositional variations of plant and soil microbial communities revealed by restricted permutational multivariate analysis of variance (Restricted PERMANOVA).

|  |  | Plant | | | | Microbe | | | | | |
| --- | --- | --- | --- | --- | --- | --- | --- | --- | --- | --- | --- |
| Plant | *Df* | R^2^ | pseudo-F | *P* | | R^2^ | | pseudo-F | | *P* | |
| Management strategy | 1 | 0.043 | 53.783 | **0.001** | | 0.005 | | 9.071 | | **0.043** | |
| N addition | 8 | 0.082 | 12.804 | **0.001** | | 0.131 | | 31.951 | | **0.001** | |
| Year | 8 | 0.058 | 9.061 | **0.001** | | 0.322 | | 78.309 | | **0.001** | |
| Management strategy × N addition | 8 | 0.026 | 4.070 | 0.164 | | 0.009 | | 2.188 | | 0.906 | |
| Management strategy×Year | 8 | 0.052 | 8.060 | **0.001** | | 0.005 | | 1.190 | | **0.002** | |
| N addition ×Year | 64 | 0.059 | 1.162 | **0.001** | | 0.035 | | 1.061 | | **0.001** | |
| Management strategy × N addition×Year | 64 | 0.033 | 0.642 | 0.337 | | 0.023 | | 0.695 | | **0.002** | |
| Residuals | 810 | 0.647 | - | - | 0.471 | | - | | - | |  |
| Total | 971 | 1.000 | - | - | 1.000 | | - | | - | |  |

Significant values (*P* < 0.05) are bolded.
